# Supplementary material for: Development of a risk score for prediction of poor treatment outcomes among patients with multidrug-resistant tuberculosis
Source: PLoS One. 2020 Jan 3;15(1):e0227100. doi: 10.1371/journal.pone.0227100 (PMC6941813; doi:10.1371/journal.pone.0227100)
Supplement: Attachment — Submitted filename: Reviewer Comments.pdf [file pone.0227100.s002.pdf]

Reviewer #: It is good research article about risk score development for prediction of poor treatment outcomes among patients with multidrug-resistant tuberculosis (MDR-TB) using routinely collected data from two large countries that have a high MDR-TB burden and are geographically, economically and epidemiologically distinct.

Further, this is the first published report on the development of a simple scoring system to predict poor treatment outcomes (death, lost to follow up or treatment failure) in patients with MDR-TB.

The authors introduced the statistical method and analysis of the poor outcomes among patients with multidrug-resistant tuberculosis, which is helpful to better understand the current methods about detection and treatment failure in patients with MDR-TB.

This developed risk score can be useful to identify and estimate the absolute risk of poor treatment outcome in patients with MDR-TB.

Thus, I suggest acceptance of this article, as this study and risk score can be helpful for the clinicians to provide a better medical care and financial and psychosocial support to patients undergoing MDR-TB treatment.
